# Robotic mitral valve repair for papillary muscle rupture

**DOI:** 10.1016/j.xjtc.2022.07.011

**Published:** 2022-08-06

**Authors:** Makoto Hashimoto, Ryuji Koshima

**Affiliations:** Department of Cardiovascular Surgery, Center of Minimally Invasive Cardiac Surgery, Sapporo Cardiovascular Clinic, Hokkaido, Japan

**Figure:**
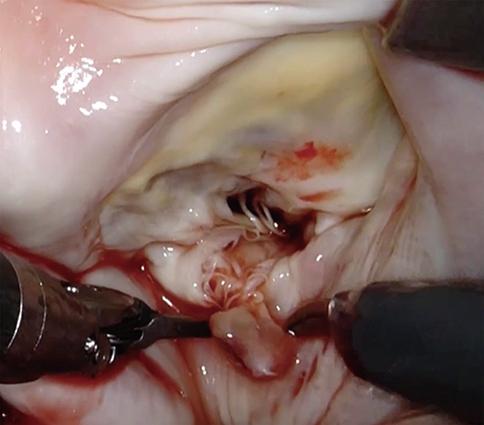
Robotic MVr for papillary muscle rupture.

**Central Message**

We present a robotically assisted mitral valve repair technique for ischemic papillary muscle rupture that facilitates favorable surgical outcomes and early recovery from this fatal complication.

Ischemic papillary muscle rupture (PMR) is a rare, life-threatening complication of acute myocardial infarction (AMI), with operative mortality reportedly between 20% and 26%.^1^^,^^2^ Some studies suggest that mitral valve repair (MVr) facilitates superior outcomes than MV replacement for such patients.^1^ In contrast, robotically assisted MVr offers the most minimally invasive approach to the surgical treatment of MV disease, and its safety and reliability have been well described.^3^^,^^4^ Considering less invasiveness and reliable results, we believe robotically assisted MVr can facilitate favorable outcomes for patients with PMR. However, only a few reports have described such cases or techniques. Herein, we describe our clinical experience and technical details of robotically assisted MVr in the case of PMR.

## Case Presentation

A 71-year-old man developed acute heart failure due to acute severe mitral regurgitation a few days after undergoing percutaneous catheter intervention for AMI of the left circumflex artery. Echocardiography revealed massive mitral regurgitation due to anterior papillary muscle head rupture, resulting in prolapse of the lateral side of the posterior MV leaflet (Video 1). The patient was barely hemodynamically stable under intravenous continuous dobutamine administration with noninvasive positive pressure ventilation. Because there was no residual coronary artery disease, the patient was considered eligible for robot-assisted surgery and was scheduled to undergo urgent robot-assisted MVr. The patient provided written informed consent. Institutional review board number and date are C22-1-02 and March 22, 2022.

## Surgical Technique

### Port Placement and Setup of Cardiopulmonary Bypass

**Figure 1:**
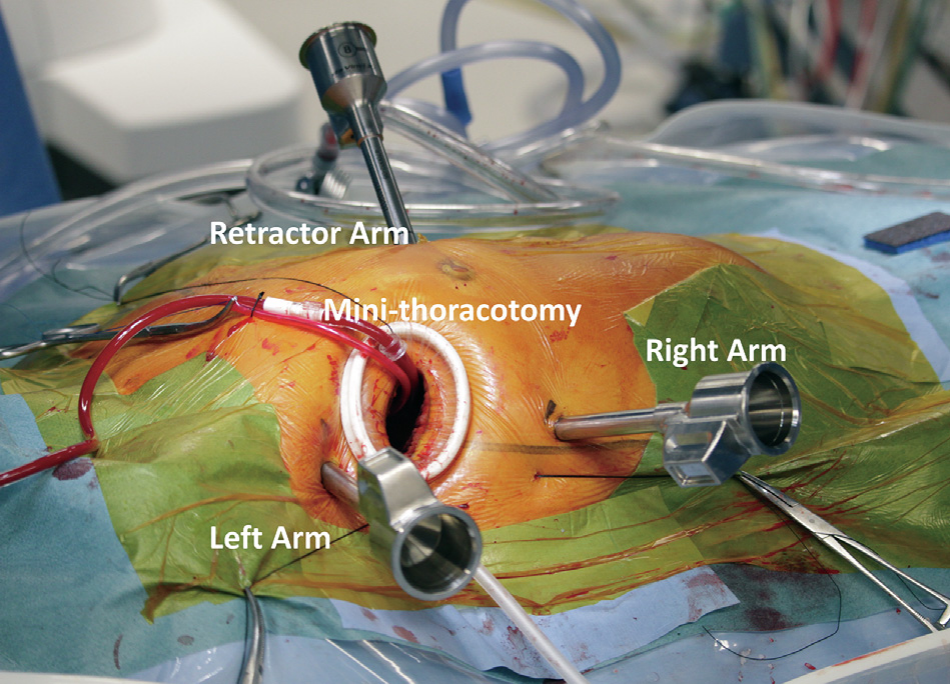
Routine port placement for robotic mitral valve repair. Camera-port is placed through the working port. Minithoracotomy and retractor arm are placed at fourth intercostal space. Left arm is placed at the third, and right arm is placed at the sixth intercostal space, respectively.

Skin incisions were made on the third (left arm, 1 cm), fourth (working port, –3 cm), and sixth (right arm, 1 cm) intercostal spaces on the right anterior axillary line (Figure 1). A 1-cm port for the atrial retractor was placed slightly medial to the midclavicle line in the fourth intercostal space. The camera was then placed through a working port. The patient-side cart of the da Vinci Xi surgical system (Intuitive Inc) was docked from the patient's left side, and cardiopulmonary bypass was initiated from the right jugular vein and femoral cannulation. An aortic root cannula was then inserted into the ascending aorta for cardioplegia. The aorta was clamped, and cardioplegia was introduced.

### MVr

**Figure 2:**
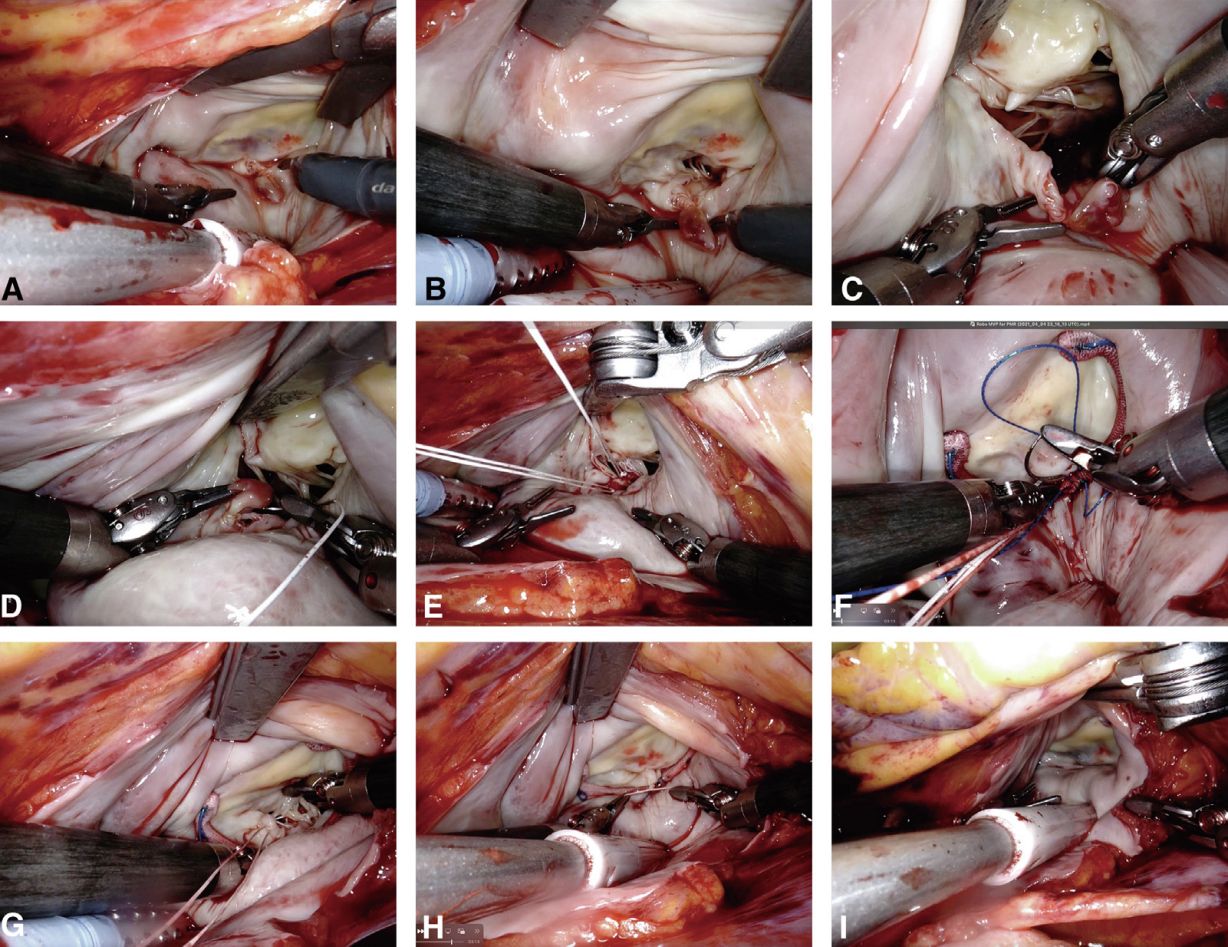
Mitral valve repair sequence for papillary muscle rupture (PMR). A, An excellent surgical view was obtained through the left atriotomy. B, A ruptured papillary muscle connected to the posterior mitral leaflet was detected. C, The robotic camera clearly showed intact anterior papillary muscle heads connected to the anterior mitral leaflet. D, The ruptured papillary muscle was sutured to the intact anterior papillary muscle by sandwiched pledget-reinforced polytetrafluoroethylene sutures. E, Both sides of the sutures were left so that it could be used as artificial chordae. F, Mitral annuloplasty was then performed by continuous suturing. G, After annuloplasty, 1 pair of the artificial chordae was attached to the lateral side of the posterior mitral leaflet. H, the artificial chordae was tied at proper length. I, A water test revealed excellent coaptation.

MVr included papillary muscle reimplantation with artificial chordae (Video 2). After left atriotomy, the atrial retractor provided an excellent view for MVr (Figure 2, *A*). One of the anterior papillary muscle heads was completely ruptured and was connected to the lateral side of the posterior MV leaflet (Figure 2, *B*). The robot camera showed an intact anterior papillary muscle with no obvious ischemic changes where the ruptured papillary muscle head could be reimplanted (Figure 2, *C*). Reimplantation was performed using sandwiched pledget-reinforced polytetrafluoroethylene sutures (Figure 2, *D*). Both sides of the sandwiched sutures were left to be used as artificial chordae to reinforce the connection between the new papillary muscle and the targeted leaflet of the MV (Figure 2, *E*). Mitral annuloplasty using a semirigid partial band (CG Future Annuloplasty Ring and Band; Medtronic) was then performed using nonabsorbable 3-0 V-Loc (Covidien) continuous sutures (Figure 2, *F*). Continuous sutures were applied from both sides of the trigon to the bottom of the mitral annulus. Finally, the artificial chordae were placed on the target leaflet. In this case, only 1 side of the artificial chordae of the sandwiched sutures facing the posterior leaflet was used as an artificial chordae. The artificial chordae were attached to the lateral side of the posterior mitral MV leaflet (Figure 2, *G*), adjusted to the optimal length, and tied (Figure 2, *H*). The water test showed excellent coaptation of the MV, with no major leak (Figure 2, *I*).

After closing the left atrium, the aortic crossclamp was released. MV function evaluated using transesophageal echocardiography showed excellent coaptation and no residual mitral regurgitation (Video 3). The remaining procedure was uneventful. The operation and cardiopulmonary bypass times were 205 minutes and 138 minutes, respectively.

The patient was discharged with no residual mitral regurgitation on postoperative day 7, without complications. A follow-up visit at the clinic 1 year postoperatively showed excellent condition with no recurrence of mitral insufficiency.

## Conclusions

This report presents our clinical experience and technical description of robotically assisted MVr for PMR. This technique of papillary muscle reimplantation with artificial chordae for reinforcement provides a reliable outcome and can be best performed using a robotic approach. The success or durability of MVr in PMR depends on the quality of the tissue where the ruptured papillary muscle head is reimplanted.^5^ The camera shows a clear and objective view of the intraventricular tissue to identify the best place for reimplantation, which we believe is key to achieving the best reliable outcome. The robot-assisted approach also allows faster recovery from life-threatening complications of AMI.

## Supplementary Data

Video 1 Preoperative transesophageal echocardiography. Video available at: https://www.jtcvs.org/article/S2666-2507(22)00422-9/fulltext.

Video 2 Mitral valve repair for papillary muscle rupture. Video available at: https://www.jtcvs.org/article/S2666-2507(22)00422-9/fulltext.

Video 3 Postoperative transesophageal and transthoracic echocardiography. Video available at: https://www.jtcvs.org/article/S2666-2507(22)00422-9/fulltext.
