# Supplementary figures and images for: Robotic mitral valve repair for papillary muscle rupture
Source: JTCVS Tech. 2022 Aug 6;15:78–80. doi: 10.1016/j.xjtc.2022.07.011 (PMC9579726; doi:10.1016/j.xjtc.2022.07.011)

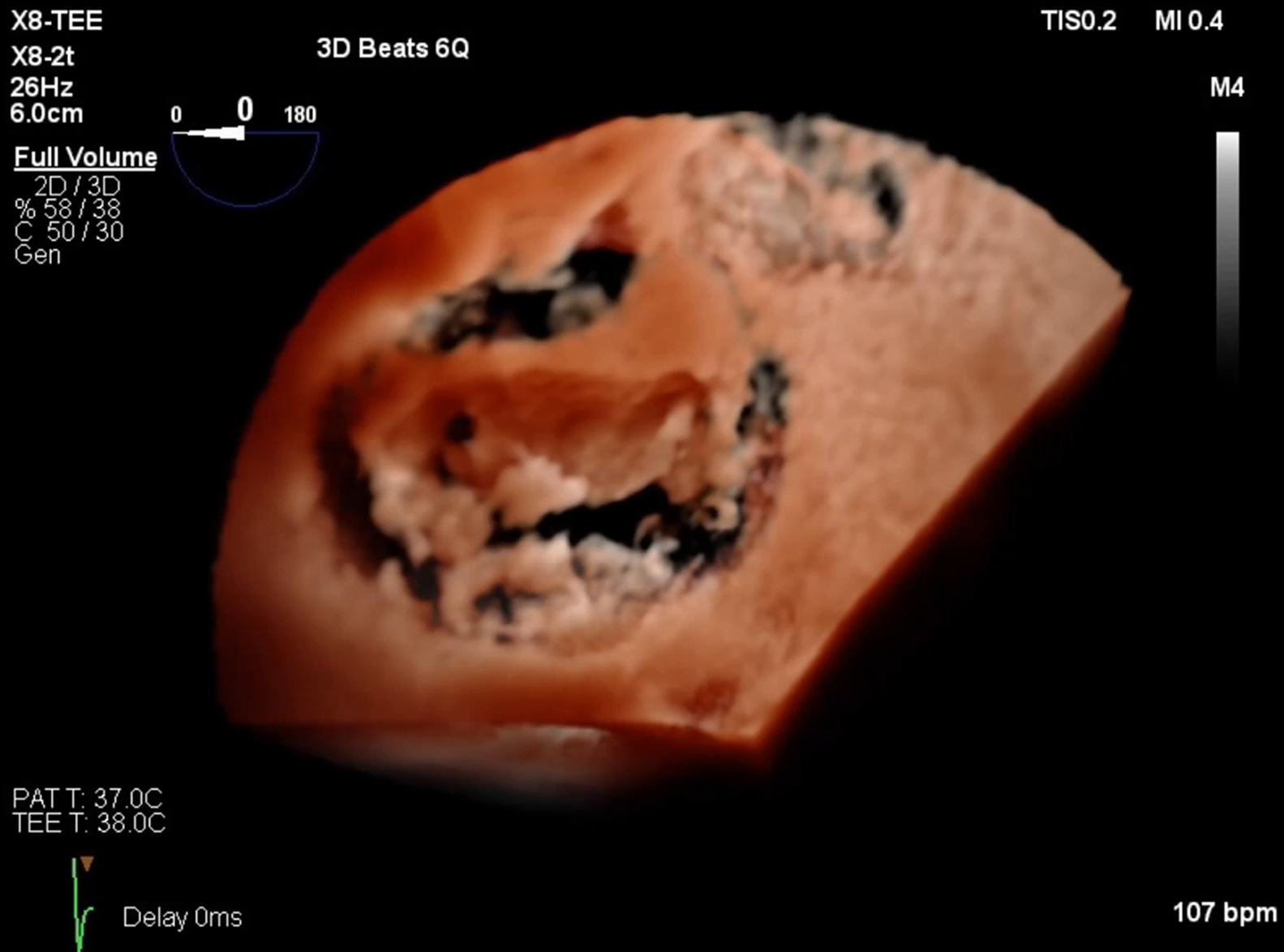

Supplement: Video 1 — Preoperative transesophageal echocardiography. Video available at: https://www.jtcvs.org/article/S2666-2507(22)00422-9/fulltext. [file fx2.jpg]

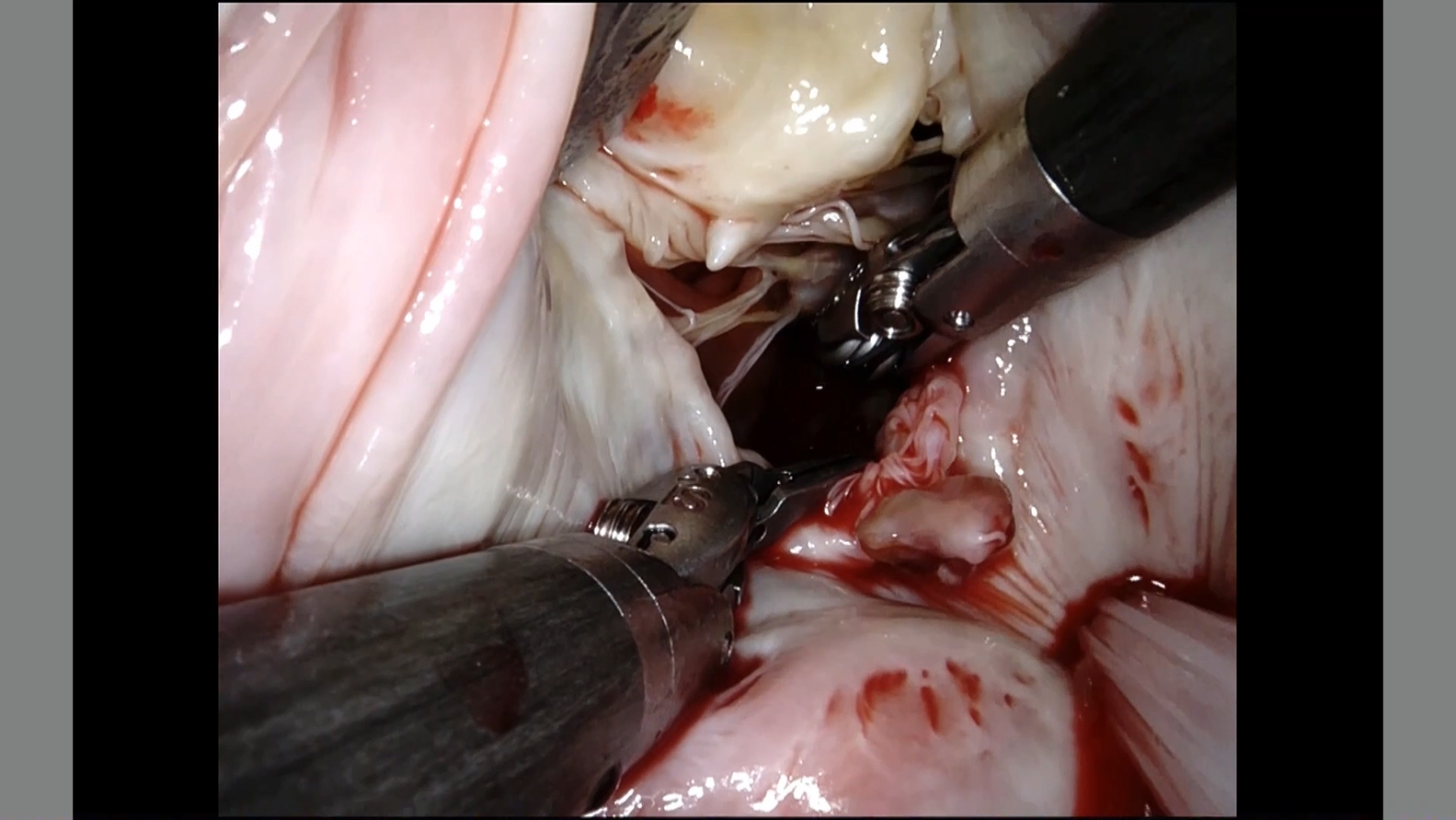

Supplement: Video 2 — Mitral valve repair for papillary muscle rupture. Video available at: https://www.jtcvs.org/article/S2666-2507(22)00422-9/fulltext. [file fx3.jpg]

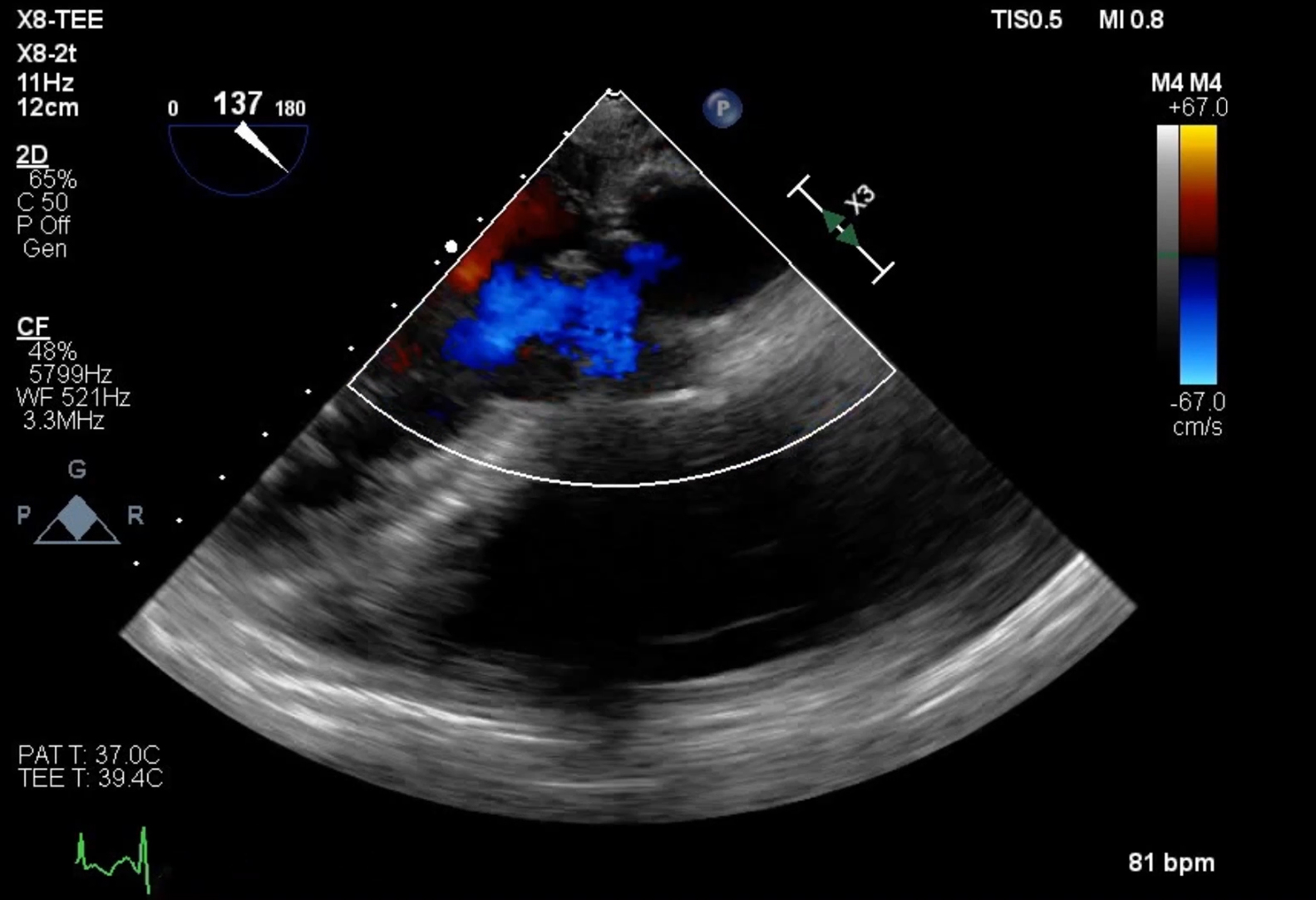

Supplement: Video 3 — Postoperative transesophageal and transthoracic echocardiography. Video available at: https://www.jtcvs.org/article/S2666-2507(22)00422-9/fulltext. [file fx4.jpg]
